# Supplementary material for: Hot spot prediction in protein-protein interactions by an ensemble system
Source: BMC Syst Biol. 2018 Dec 31;12(Suppl 9):132. doi: 10.1186/s12918-018-0665-8 (PMC6311905; doi:10.1186/s12918-018-0665-8)
Supplement: Supplementary file 2 — All relevant MATLAB code. A simple Matlab implement of our predictor. (ZIP 19,742 kb) [file 12918_2018_665_MOESM2_ESM.zip › Additional file 2/Read ASA/ASA website/BID/1FOEB.htm]

 NetSurfP - Protein Surface Accessibility and Secondary Structure Predictions

  

|  |  |  |
| --- | --- | --- |
|  |  | NetSurfP - Protein Surface Accessibility and Secondary Structure Predictions  Technical University of Denmark |

  


---

```
# For publication of results, please cite:
# A generic method for assignment of reliability scores applied to solvent accessibility predictions.
# Bent Petersen, Thomas Nordahl Petersen, Pernille Andersen, Morten Nielsen and Claus Lundegaard
# BMC Structural Biology 2009, 9:51 doi:10.1186/1472-6807-9-51
#
# Column 1: Class assignment - B for buried or E for Exposed - Threshold: 25% exposure, but not based on RSA
# Column 2: Amino acid
# Column 3: Sequence name
# Column 4: Amino acid number
# Column 5: Relative Surface Accessibility - RSA
# Column 6: Absolute Surface Accessibility
# Column 7: Z-fit score for RSA prediction
# Column 8: Probability for Alpha-Helix
# Column 9: Probability for Beta-strand
# Column 10: Probability for Coil
E M  1FOEB                  1    0.663 132.586  -0.529   0.003   0.003   0.994
E Q  1FOEB                  2    0.506  90.354   1.128   0.004   0.138   0.858
E A  1FOEB                  3    0.415  45.722   1.777   0.003   0.718   0.279
B I  1FOEB                  4    0.064  11.785   1.008   0.002   0.816   0.182
B K  1FOEB                  5    0.154  31.637   1.390   0.001   0.900   0.099
B C  1FOEB                  6    0.027   3.791   1.132   0.000   0.983   0.017
B V  1FOEB                  7    0.023   3.520   1.448   0.000   0.983   0.017
B V  1FOEB                  8    0.020   3.105   1.325   0.001   0.959   0.040
B V  1FOEB                  9    0.027   4.119   0.926   0.001   0.900   0.099
B G  1FOEB                 10    0.047   3.723  -0.200   0.004   0.420   0.576
B D  1FOEB                 11    0.208  29.987  -0.505   0.005   0.045   0.951
B G  1FOEB                 12    0.235  18.479  -1.212   0.018   0.019   0.964
B A  1FOEB                 13    0.200  22.018  -1.288   0.053   0.043   0.903
B V  1FOEB                 14    0.048   7.347   0.186   0.268   0.043   0.689
B G  1FOEB                 15    0.037   2.943  -0.797   0.354   0.048   0.598
B K  1FOEB                 16    0.093  19.068   0.151   0.660   0.049   0.291
B T  1FOEB                 17    0.060   8.391  -0.230   0.701   0.107   0.192
B C  1FOEB                 18    0.038   5.363  -0.074   0.779   0.100   0.120
B L  1FOEB                 19    0.032   5.914   0.207   0.870   0.077   0.053
B L  1FOEB                 20    0.023   4.230   0.622   0.807   0.137   0.056
B I  1FOEB                 21    0.116  21.460   0.413   0.807   0.137   0.056
B S  1FOEB                 22    0.092  10.759   0.999   0.807   0.137   0.056
B Y  1FOEB                 23    0.083  17.694   0.116   0.779   0.100   0.120
B T  1FOEB                 24    0.063   8.710   0.685   0.660   0.049   0.291
E T  1FOEB                 25    0.527  73.150   1.051   0.430   0.016   0.555
E N  1FOEB                 26    0.574  84.078  -0.499   0.115   0.016   0.868
E A  1FOEB                 27    0.525  57.910   1.383   0.053   0.043   0.903
B F  1FOEB                 28    0.065  13.005   0.168   0.018   0.047   0.935
E P  1FOEB                 29    0.431  61.116   1.497   0.018   0.047   0.935
E G  1FOEB                 30    0.706  55.546  -0.342   0.018   0.047   0.935
E E  1FOEB                 31    0.655 114.428   0.065   0.052   0.084   0.864
B Y  1FOEB                 32    0.226  48.232   0.754   0.064   0.216   0.721
E I  1FOEB                 33    0.471  87.098   0.681   0.056   0.142   0.802
B P  1FOEB                 34    0.170  24.151   0.256   0.118   0.150   0.732
B T  1FOEB                 35    0.160  22.234   0.048   0.231   0.330   0.439
B V  1FOEB                 36    0.120  18.459   0.897   0.252   0.423   0.325
B F  1FOEB                 37    0.167  33.597  -0.879   0.252   0.423   0.325
B D  1FOEB                 38    0.187  26.990   0.009   0.159   0.516   0.325
B N  1FOEB                 39    0.298  43.569  -0.573   0.079   0.592   0.329
B Y  1FOEB                 40    0.108  23.015   0.741   0.018   0.846   0.136
E S  1FOEB                 41    0.303  35.558   1.098   0.001   0.900   0.099
E A  1FOEB                 42    0.269  29.699   1.361   0.001   0.959   0.040
E N  1FOEB                 43    0.396  57.930   1.742   0.001   0.959   0.040
B V  1FOEB                 44    0.064   9.775   1.149   0.001   0.959   0.040
E M  1FOEB                 45    0.464  92.886   0.718   0.001   0.959   0.040
B V  1FOEB                 46    0.148  22.732   0.644   0.001   0.900   0.099
E D  1FOEB                 47    0.698 100.524   0.367   0.004   0.085   0.910
E G  1FOEB                 48    0.788  62.047   0.165   0.003   0.003   0.994
E K  1FOEB                 49    0.528 108.630   1.343   0.003   0.718   0.279
E P  1FOEB                 50    0.413  58.605   2.105   0.001   0.959   0.040
B V  1FOEB                 51    0.042   6.471   1.228   0.006   0.962   0.032
E N  1FOEB                 52    0.296  43.276   2.461   0.006   0.962   0.032
B L  1FOEB                 53    0.024   4.468   1.033   0.006   0.962   0.032
B G  1FOEB                 54    0.113   8.893   1.106   0.011   0.918   0.071
B L  1FOEB                 55    0.024   4.431   0.997   0.011   0.918   0.071
B W  1FOEB                 56    0.079  19.072   0.917   0.021   0.756   0.223
B D  1FOEB                 57    0.064   9.208   0.174   0.022   0.552   0.426
B T  1FOEB                 58    0.099  13.787  -0.057   0.056   0.142   0.802
B A  1FOEB                 59    0.139  15.296  -0.359   0.113   0.043   0.844
E G  1FOEB                 60    0.256  20.163  -0.446   0.181   0.016   0.803
B Q  1FOEB                 61    0.224  39.989   0.489   0.430   0.016   0.555
E E  1FOEB                 62    0.409  71.452   0.096   0.522   0.016   0.462
E D  1FOEB                 63    0.436  62.784   0.865   0.522   0.016   0.462
B Y  1FOEB                 64    0.093  19.917   0.245   0.522   0.016   0.462
E D  1FOEB                 65    0.465  67.050   1.117   0.622   0.015   0.363
E R  1FOEB                 66    0.339  77.654   0.071   0.522   0.016   0.462
B L  1FOEB                 67    0.100  18.273  -0.124   0.522   0.016   0.462
B R  1FOEB                 68    0.172  39.502   0.794   0.430   0.016   0.555
E P  1FOEB                 69    0.462  65.544   0.004   0.455   0.046   0.498
B L  1FOEB                 70    0.209  38.305  -0.289   0.386   0.097   0.517
B S  1FOEB                 71    0.140  16.396  -0.512   0.307   0.165   0.527
B Y  1FOEB                 72    0.086  18.485   0.465   0.199   0.152   0.649
E P  1FOEB                 73    0.357  50.672   0.140   0.113   0.087   0.800
E Q  1FOEB                 74    0.355  63.474  -0.978   0.053   0.043   0.903
B T  1FOEB                 75    0.030   4.147  -0.066   0.018   0.088   0.893
B D  1FOEB                 76    0.143  20.563  -0.237   0.005   0.336   0.660
B V  1FOEB                 77    0.024   3.643   0.683   0.001   0.900   0.099
B F  1FOEB                 78    0.017   3.392   1.340   0.001   0.959   0.040
B L  1FOEB                 79    0.017   3.186   1.625   0.001   0.959   0.040
B I  1FOEB                 80    0.019   3.515   1.729   0.000   0.983   0.017
B C  1FOEB                 81    0.024   3.313   1.115   0.001   0.959   0.040
B F  1FOEB                 82    0.057  11.520   1.178   0.001   0.959   0.040
B S  1FOEB                 83    0.072   8.474  -0.869   0.001   0.900   0.099
B L  1FOEB                 84    0.051   9.356   0.727   0.004   0.420   0.576
E V  1FOEB                 85    0.339  52.181   0.336   0.005   0.045   0.951
E S  1FOEB                 86    0.348  40.797   1.051   0.018   0.019   0.964
E P  1FOEB                 87    0.445  63.075   1.407   0.600   0.003   0.397
E A  1FOEB                 88    0.648  71.377   1.267   0.406   0.004   0.590
B S  1FOEB                 89    0.072   8.438  -0.199   0.600   0.003   0.397
B F  1FOEB                 90    0.059  11.942   0.081   0.923   0.002   0.076
E E  1FOEB                 91    0.554  96.784   1.582   0.923   0.002   0.076
E N  1FOEB                 92    0.434  63.611   1.188   0.923   0.002   0.076
B V  1FOEB                 93    0.022   3.320   0.979   0.970   0.001   0.030
B R  1FOEB                 94    0.283  64.807   0.967   0.970   0.001   0.030
E A  1FOEB                 95    0.651  71.696   1.097   0.970   0.001   0.030
E K  1FOEB                 96    0.263  54.058   1.580   0.970   0.001   0.030
B W  1FOEB                 97    0.045  10.823   0.472   0.970   0.001   0.030
E Y  1FOEB                 98    0.257  54.964   1.520   0.970   0.001   0.030
E P  1FOEB                 99    0.532  75.420   1.469   0.988   0.000   0.012
B E  1FOEB                100    0.087  15.181  -0.017   0.970   0.001   0.030
B V  1FOEB                101    0.047   7.270   0.577   0.970   0.001   0.030
E R  1FOEB                102    0.525 120.202   1.519   0.923   0.002   0.076
E H  1FOEB                103    0.616 112.032   1.094   0.782   0.003   0.216
E H  1FOEB                104    0.298  54.206   0.047   0.406   0.004   0.590
B C  1FOEB                105    0.111  15.641  -0.907   0.016   0.005   0.979
E P  1FOEB                106    0.734 104.155   0.213   0.003   0.003   0.994
E N  1FOEB                107    0.600  87.899   0.776   0.005   0.015   0.979
B T  1FOEB                108    0.033   4.633   1.255   0.004   0.138   0.858
B P  1FOEB                109    0.093  13.225   0.898   0.004   0.616   0.381
B I  1FOEB                110    0.044   8.177   0.912   0.001   0.959   0.040
B I  1FOEB                111    0.014   2.627   1.745   0.000   0.983   0.017
B L  1FOEB                112    0.015   2.692   1.686   0.001   0.959   0.040
B V  1FOEB                113    0.015   2.367   1.397   0.001   0.900   0.099
B G  1FOEB                114    0.021   1.637   0.048   0.004   0.616   0.381
B T  1FOEB                115    0.062   8.669   0.015   0.022   0.359   0.619
B K  1FOEB                116    0.159  32.665   0.415   0.020   0.205   0.775
B L  1FOEB                117    0.150  27.447   0.226   0.018   0.088   0.893
B D  1FOEB                118    0.155  22.307  -0.594   0.053   0.043   0.903
B L  1FOEB                119    0.113  20.727   0.277   0.181   0.016   0.803
E R  1FOEB                120    0.508 116.309   0.724   0.406   0.004   0.590
E D  1FOEB                121    0.656  94.558  -0.082   0.502   0.002   0.495
E D  1FOEB                122    0.399  57.438  -0.137   0.502   0.002   0.495
E K  1FOEB                123    0.437  89.850  -0.577   0.717   0.014   0.269
E D  1FOEB                124    0.623  89.717  -0.303   0.622   0.015   0.363
E T  1FOEB                125    0.296  41.083  -0.178   0.622   0.015   0.363
B I  1FOEB                126    0.268  49.580  -0.723   0.717   0.014   0.269
E E  1FOEB                127    0.535  93.395  -0.378   0.717   0.014   0.269
E K  1FOEB                128    0.487 100.217  -0.071   0.717   0.014   0.269
B L  1FOEB                129    0.216  39.531  -1.272   0.717   0.014   0.269
E K  1FOEB                130    0.480  98.798  -0.799   0.717   0.014   0.269
E E  1FOEB                131    0.647 113.013  -0.091   0.502   0.002   0.495
E K  1FOEB                132    0.526 108.157  -0.912   0.321   0.003   0.675
E K  1FOEB                133    0.526 108.198  -0.906   0.115   0.016   0.868
E L  1FOEB                134    0.469  85.929  -0.590   0.018   0.047   0.935
E T  1FOEB                135    0.483  66.937   0.256   0.018   0.088   0.893
E P  1FOEB                136    0.429  60.918   0.504   0.004   0.197   0.799
B I  1FOEB                137    0.034   6.216  -0.241   0.004   0.138   0.858
E T  1FOEB                138    0.352  48.795   1.476   0.018   0.047   0.935
E Y  1FOEB                139    0.387  82.702   0.986   0.923   0.002   0.076
E P  1FOEB                140    0.711 100.863   1.022   0.970   0.001   0.030
E Q  1FOEB                141    0.321  57.348   1.289   0.970   0.001   0.030
B G  1FOEB                142    0.022   1.763   0.038   0.970   0.001   0.030
E L  1FOEB                143    0.350  64.048   1.897   0.970   0.001   0.030
E A  1FOEB                144    0.565  62.263   1.870   0.970   0.001   0.030
B M  1FOEB                145    0.093  18.609   1.383   0.970   0.001   0.030
B A  1FOEB                146    0.016   1.796   0.509   0.970   0.001   0.030
E K  1FOEB                147    0.661 135.947   1.685   0.970   0.001   0.030
E E  1FOEB                148    0.633 110.585   1.319   0.923   0.002   0.076
B I  1FOEB                149    0.208  38.406   1.077   0.694   0.003   0.303
E G  1FOEB                150    0.599  47.165  -0.378   0.053   0.005   0.942
B A  1FOEB                151    0.206  22.690   0.222   0.018   0.019   0.964
B V  1FOEB                152    0.091  13.971   0.791   0.020   0.205   0.775
E K  1FOEB                153    0.227  46.673   1.234   0.023   0.655   0.322
B Y  1FOEB                154    0.051  10.877   0.524   0.018   0.846   0.136
B L  1FOEB                155    0.053   9.686   0.478   0.018   0.846   0.136
B E  1FOEB                156    0.053   9.242  -0.282   0.018   0.846   0.136
B C  1FOEB                157    0.025   3.468   0.746   0.079   0.592   0.329
B S  1FOEB                158    0.069   8.028   0.176   0.135   0.317   0.548
B A  1FOEB                159    0.049   5.378  -1.822   0.386   0.097   0.517
E L  1FOEB                160    0.264  48.283   0.450   0.257   0.016   0.727
E T  1FOEB                161    0.460  63.788   0.512   0.115   0.016   0.868
E Q  1FOEB                162    0.521  92.997  -0.228   0.321   0.003   0.675
E R  1FOEB                163    0.505 115.737   1.468   0.406   0.004   0.590
E G  1FOEB                164    0.260  20.493   1.002   0.181   0.016   0.803
B L  1FOEB                165    0.023   4.156   1.363   0.923   0.002   0.076
E K  1FOEB                166    0.419  86.168   2.307   0.970   0.001   0.030
E T  1FOEB                167    0.443  61.458   2.109   0.970   0.001   0.030
B V  1FOEB                168    0.031   4.719   1.219   0.970   0.001   0.030
B F  1FOEB                169    0.023   4.596   1.359   0.988   0.000   0.012
E D  1FOEB                170    0.322  46.386   2.221   0.988   0.000   0.012
E E  1FOEB                171    0.348  60.813   2.211   0.988   0.000   0.012
B A  1FOEB                172    0.040   4.430   0.858   0.970   0.001   0.030
B I  1FOEB                173    0.037   6.919  -0.375   0.970   0.001   0.030
E R  1FOEB                174    0.444 101.584   2.021   0.923   0.002   0.076
E A  1FOEB                175    0.469  51.706   1.997   0.923   0.002   0.076
B V  1FOEB                176    0.093  14.263   0.122   0.782   0.003   0.216
E L  1FOEB                177    0.394  72.196  -0.834   0.109   0.005   0.886

Explain the output.  Go back.
```

---
